# Nucleocapsid Protein of SARS-CoV-2 Upregulates RANTES Expression in A172 Glioblastoma Cells

**DOI:** 10.3390/molecules30051066

**Published:** 2025-02-26

**Authors:** Bakhytgul Gadilgereyeva, Zhanar Kunushpayeva, Mira Abdrakhmanova, Aizere Khassenova, Nail Minigulov, Timo Burster, Olena Filchakova

**Affiliations:** Biology Department, School of Sciences and Humanities, Nazarbayev University, Kabanbay Batyr ave., 53, Astana 010000, Kazakhstanmira.abdrakhmanova@alumni.nu.edu.kz (M.A.); aizere.khassenova@nu.edu.kz (A.K.); nail.minigulov@nu.edu.kz (N.M.); timo.burster@nu.edu.kz (T.B.)

**Keywords:** coronaviruses, SARS-CoV-2, nucleocapsid protein, RANTES, glioblastoma

## Abstract

SARS-CoV-2, the pathogenic virus that induces COVID-19 disease, contains four structural proteins in its virion. The nucleocapsid (N) protein is one of the four structural proteins that play a crucial role in the assembly of viral RNA into ribonucleoprotein. In addition, the N protein contributes to viral pathogenesis. One of the functions attributed to the N protein is the triggering of cytokine release by lung epithelial cells, macrophages, and monocytes. This study addresses the cellular effects of the N protein of SARS-CoV-2 on cells of glial origin. We report the upregulation of the RANTES chemokine in A172 glioblastoma cells at both the mRNA and protein levels in response to exposure to SARS-CoV-2 nucleocapsid protein. The N protein did not have an effect on cell viability and cell migration.

## 1. Introduction

Coronavirus disease 2019 (COVID-19) is a pandemic that emerged at the end of 2019 and had a profound global effect. The disease is caused by a novel virus, Severe Acute Respiratory Syndrome Coronavirus 2 (SARS-CoV-2). The virus is a positive-sense, single-stranded RNA virus, containing 29,903 nucleotides. The viral genome codes for approximately 30 mature proteins and contains four structural protein genes, namely the spike surface glycoprotein gene, the envelope gene, the matrix gene, and the nucleocapsid (N) phosphoprotein gene [1]. The functions of some, but not all, protein products of the viral genome are characterized. However, there is still a significant gap in our understanding of the full functional range of viral-derived proteins and the host-specific responses that occur upon exposure to such viral proteins. Thus, the function of SARS-CoV-2’s N structural protein was investigated with respect to its potential to upregulate cytokines in glioblastoma cells.

One significant cause of mortality associated with SARS-CoV-2 infection is acute respiratory distress syndrome (ARDS) due to a massive cytokine storm in lung tissue. The lung epithelial cells upregulate proinflammatory cytokines in response to viral exposure. IL-6 is one of the proinflammatory cytokines that was shown to be upregulated within the lung cytokine storm in COVID-19 patients [2]. Previous studies demonstrated that N protein, one of four structural proteins found in coronaviruses, can trigger IL-6 release. For example, lung epithelial cells upregulate IL-6 expression following transfection with SARS-CoV N protein-coding plasmid [3]. This effect was through the induction of NF-kB transcription factor. In addition, the SARS-CoV-2 N protein induces IL-6 expression in monocytes and macrophages [4].

Besides its effect on lung tissue, there are indications of neurological disorders associated with COVID-19 [5,6,7]. Anosmia (loss of smell) is by far one of the most prominent symptoms suggesting the involvement of the nervous system in COVID-19 pathogenesis [8]. A recent study by Meinhardt et al. wherein post-mortem tissues were analyzed by quantitative real-time PCR (RTR-qPCR) [9] demonstrated the presence of viral RNA in olfactory mucosa in 20 out of 30 samples. Their study suggests the olfactory route is used by the virus to enter the CNS. The cellular consequences of viral entry to the nervous system, as well as the mechanism of cellular entry and types of affected cells, are not well understood. It has been established that SARS-CoV’s and SARS-CoV-2’s N proteins upregulate cytokines (particularly, IL-6) in different cells. Within this project, we aimed to elucidate the effect of SARS-CoV-2’s N protein on cytokine release from glioblastoma cells. We hypothesized that the SARS-CoV-2 N protein upregulates cytokine IL-6 in the cells of glial origin.

Contrary to our expectations regarding IL-6 upregulation, we failed to detect changes in IL-6 expression. The most significant observation was the upregulation of the RANTES chemokine in the glioblastoma cell line A172. This effect was cell-type-specific and observed at both the protein and mRNA levels. The N protein did not influence the migration of A172 cells and did not negatively influence cell viability. In addition, the N protein provoked the upregulation of cathepsin S in A172 cells.

## 2. Results

### 2.1. N Protein Upregulates RANTES Expression

**Figure 1:**
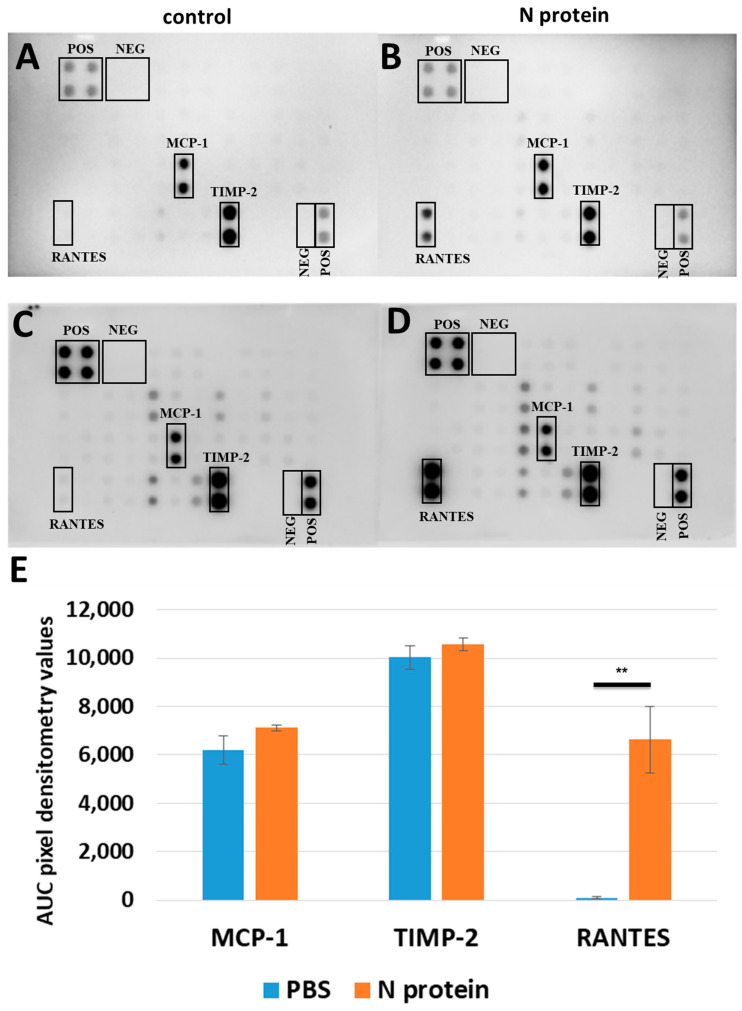
Representative cytokine array results for A172 cell line. A172 cells were treated either with PBS (**A**,**C**) or with 1 μg/mL of recombinant N protein (**B**,**D**) for 48 h. The area under the curve (AUC) densitometry values for most expressed cytokines on both arrays were analyzed by image J (https://imagej.net/ij/); normalized data are shown (**E**); error bars correspond to SEM; n = 4. There was a non-significant difference for MCP-1 and TIMP-2 pairs; for RANTES pair, *p*-value is 0.006 determined by Student’s *t*-test. ** corresponds to a *p*-value less than 0.01.

**Figure 2:**
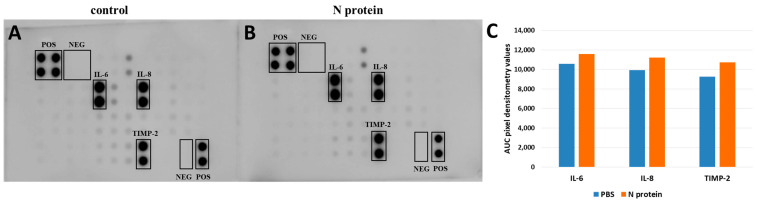
Representative cytokine array results for U87 cell line. U87 cells were treated either with PBS (**A**) or with 1 μg/mL of recombinant N protein (**B**) for 48 h. (**C**) The area under the curve (AUC) densitometry values were analyzed using image J, and normalized data are shown.

In order to test our hypothesis of cytokine upregulation in response to N protein exposure, we treated A172 and U87 cells—two glioblastoma cell lines—with recombinant N protein at 1 μg/mL concentration for 48 h. This concentration and time window selection was in accordance with previously published results which demonstrated the upregulation of IL-6 cytokine [4]. The results demonstrated that different cell lines express different sets of cytokines, and that the N protein has a cell-specific effect, leading to a significant upregulation of RANTES in A172 cells (Figure 1 and Figure 2). Thus, A172 cells express MCP-1 and TIMP-2 at high levels, as compared to other cytokines, both before and after the stimulation (Figure 1), while U87 cells express IL-6, IL-8, and TIMP-2 cytokines at high levels, both before and after the stimulation (Figure 2). The average AUC pixel densitometry values for MCP-1 cytokine in A172 cells were 5041.24 and 7365.53 for control, and 7212.79 and 7019.17 after N protein stimulation. The average AUC pixel densitometry values for TIMP-2 cytokine in A172 cells were 9057.99 and 10,999.514 for control, and 10,071.11 and 11,063.28 following stimulation.

The most striking and notable observation was the upregulation of RANTES/CCL-5 chemokine in A172 cells. Following N protein exposure, AUC pixel values for RANTES were measured to be 3940.54 and 9318.48. The RANTES signal was not detectable on one blot, while the second blot produced a low value of 186.23 in the PBS-treated sample.

Surprisingly, and contrary to our expectations, IL-6 was not upregulated in response to N protein exposure (Figure 1 and Figure 2). Moreover, A172 cells failed to express IL-6 at detectable levels in both stimulated and unstimulated conditions. The average AUC pixel densitometry values for cytokines expressed in U87 cells were the following: IL-6—10,567.25 for control and 11,578.28 following stimulation; IL-8—9929.18 for control and 11,198.11 after stimulation; TIMP-2—9261.78 for control and 10,734.05 for stimulated cells. Unlike A172 cells, U87 cells failed to express RANTES at a detectable level (Figure 2).

**Figure 3:**
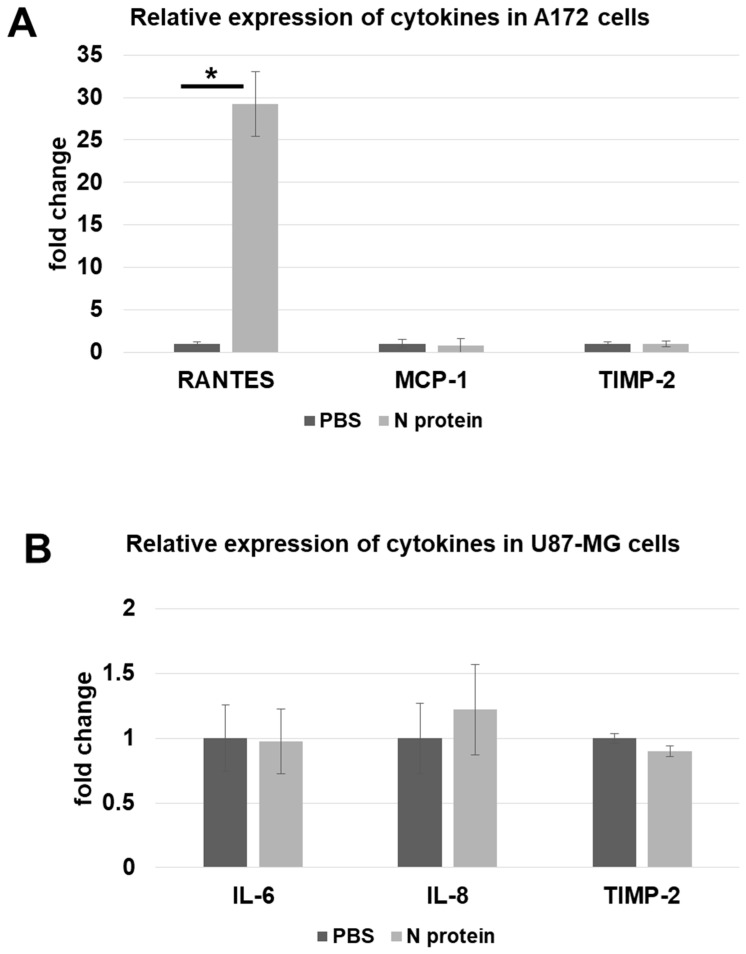
Relative change in expression for cytokines as measured by RT-qPCR. The fold change expression is shown, and the data were normalized to GAPD control. (**A**) Cytokines expressed by A172 cells are shown—RANTES, MCP-1, and TIMP-2. The number of biological replicates are 4, 5, and 5 for RANTES, MCP-1, and TIMP-2, respectively. A *p*-value of 0.02 was calculated for the RANTES pair. (**B**) The cytokines tested on U87 are IL-6, IL-8, and TIMP-2. The data from 4, 8, and 5 biological replicates for IL-6, IL-8, and TIMP-2, respectively, are shown. Three technical replicates were used for each biological replicate. The ΔΔCt method was used to analyze the data; error bars represent standard errors. * *p*-value less than 0.05.

In order to confirm the upregulation of RANTES in A172 cells at the mRNA level, we conducted an RT-qPCR experiment (Figure 3). The data demonstrate a significant upregulation of RANTES mRNA in A172 cells following N protein application. A 29.2-fold increase was observed. Unlike RANTES, MCP-1 and TIMP-2 were not affected by N protein treatment. The levels of IL-6, IL-8, and TIMP-2 in U87 cells did not change following N protein exposure.

### 2.2. N Protein Has No Effect on Cell Viability and Cell Migration

**Figure 4:**
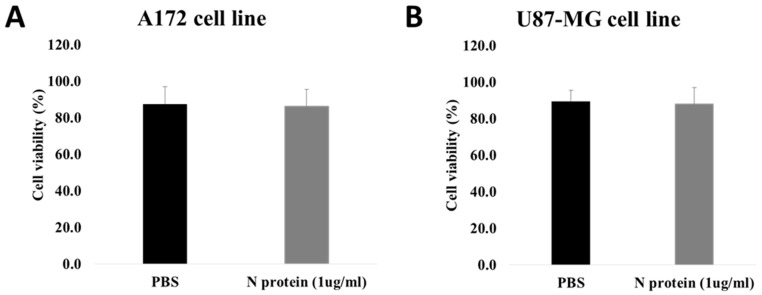
Graphical representation of the cell viability measurements. The A172 cells (**A**) and U87 cells (**B**) were treated with 1 µg/mL of N protein for 48 h. PBS was used as a control. The data are from five independent experiments. Student’s *t*-test was used for statistical analysis, and a non-significant difference was found between the control and treatment groups. Error bars represent standard deviation.

We evaluated the effect of SARS-CoV-2’s N protein on cell viability through PI staining using flow cytometry. There was no statistically significant difference in the percentage of live cells between the PBS-treated group and the N protein-treated group (Figure 4). For A172 cells, the average cell viability for the PBS group was 87.5 ± 9.4%, while for the experimental group, it was 86.3 ± 9.3%. For U87 cells, the PBS group average cell viability equated to 89.4 ± 6.2%, and it was found to be 88.1 ± 8.9% for the N protein treatment group. These results suggest that N protein (at a tested concentration) does not possess cytotoxicity.

**Figure 5:**
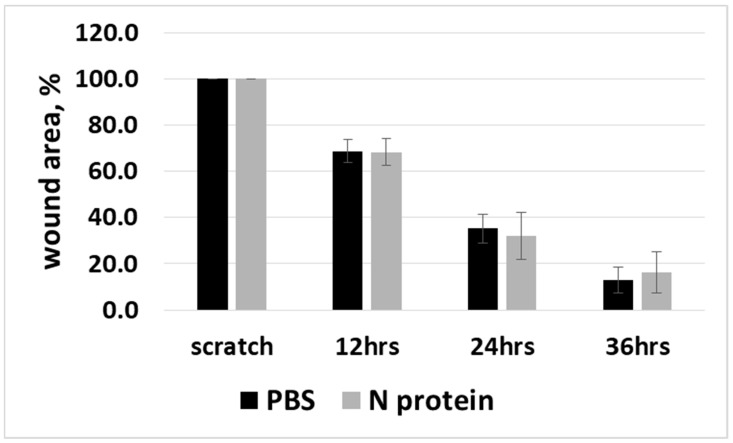
Scratch wound healing assay to determine cell migration. Wound healing assays were performed at 10 min, 12 h, 24 h, and 36 h following the scratch in untreated (PBS) control cells and A172 cells treated with 1 µg/mL of N protein. The images were analyzed using the Image J software. The graph shows the average values of four independent experiments with n = 16 for the PBS group and n = 17 for the N protein group. Error bars represent standard errors. Student’s *t*-test was used for statistical analysis, and a non-significant difference was detected between the control and experimental groups.

Considering that the RANTES cytokine was shown to enable cell migration [10], we tested the ability of N protein to induce cell migration in A172 cells. A wound-healing scratch assay was used. The results showed no difference in cell migration between the PBS control group and the N protein treatment group (Figure 5).

### 2.3. N Protein Provokes an Increase in Cathepsin S in A172 Cells

**Figure 6:**
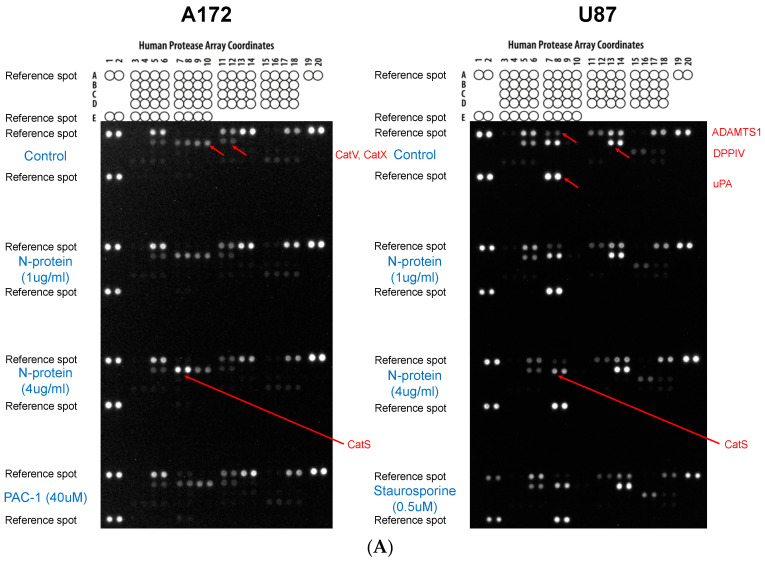

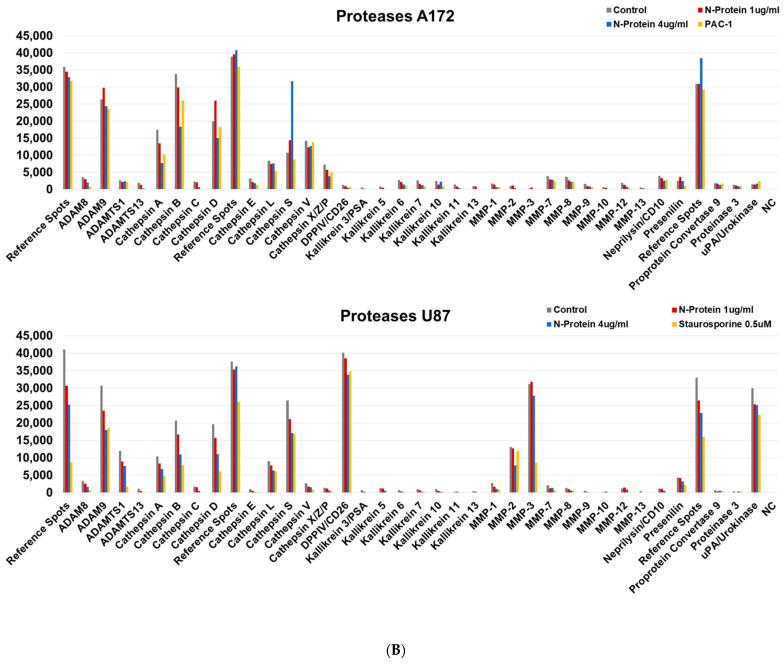
Representative protease array on A172 and U87 cell lines. The cells were cultured with either 1 µg/mL or 4 µg/mL of N protein for 24 h. Afterwards a panel of 35 different proteases was analyzed by using the Protease Profiler Array to assess the changes in protease levels. The experiment was repeated three times. The images of the arrays (**A**) and the quantitative analysis of the array results (**B**) are shown. PBS was used as a negative control; PAC-1 [40 µM] and staurosporine [0.5 µM] were used as positive controls for A172 and U87 cells, respectively. One representative blot is shown (A172 (n = 3), and U87 (n = 2) for control vs. 1 or 4 µg/mL N protein). Red arrows point to a corresponding proteases shown on the right side of the array.

Next, we investigated the impact of N protein on protease levels in A172 and U87 cells, and found that the N protein affects the expression of cathepsin S in A172 in a dose-dependent fashion, with a more significant effect observed at an N protein concentration of 4 µg/mL (refer to Figure 6). Interestingly, this effect was most likely specific to A172 cells since U87 cells did not exhibit an increase in cathepsin S expression. It was also observed that the level of cathepsin S is higher in non-stimulated U87MG cells than in A172 cells, as published in [11]. Interestingly, urokinase-type plasminogen activator (uPA), matrix metalloproteinase (MMP)-3, MMP-2, a disintegrin and metalloproteinase with thrombospondin motifs 1 (ADAMTS1), and dipeptidyl peptidase-4 (DPPIV) were exclusively found in U87 cells, while cathepsin V and X (CatX) were only detected in A172 cells. This finding regarding CatX aligns with previous reports in the literature [11].

## 3. Discussion

Arising in 2019, the COVID-19 pandemic caused by the SARS-CoV-2 virus circulated worldwide, resulting in the transmission of the disease to hundreds of millions of people around the globe. To this day, the majority of mechanisms by which productive viral infection occurs, resulting in severe cases of disease, are not fully understood. Severe forms of COVID-19 are associated with a cytokine storm, which is an uncontrolled inflammatory acute reaction of the immune system eliciting an overproduction of cytokines [12,13]. Multiple studies show high levels of proinflammatory cytokines such as IL-1, TNF, IFNs, IL-6, IL-8, IL-18, IL-17α, G-CSF, and GM-CSF, as well as chemokines, such as MCP-1, IP-10, MIP-1α, CXCL10, CCL2, CCL4, CCL14, CCL19, and CCL25 in severe cases of COVID-19 [14,15,16]. Among the upregulated proinflammatory cytokines, IL-6 plays a significant role [15,17]. Hyperinflammation followed by a subsequent cytokine storm is associated with the high production of IL-6, which, in turn, is correlated with multiple organ failure in patients affected by SARS-CoV-2 [18,19].

In this research, the hypothesis of IL-6 upregulation in glial cells in response to SARS-CoV-2 N protein exposure was investigated. Based on the previously published data, which demonstrated an increase in the level of IL-6 in A549 cells following transfection with SARS-CoV N protein-coding plasmid [3], as well as the upregulation of IL-6 in human monocytes and macrophages following SARS-CoV-2 N protein exposure [4], and considering the high degree of similarity between SARS-CoV and SARS-CoV-2 N proteins [20], we expected other cells to exhibit an increase in IL-6 level as well. When U87 and A172 glioblastoma cells were tested, we failed to detect IL-6 upregulation. This suggests that the effect of cytokine upregulation is cell-type-specific, which necessitates further investigation into the involved molecular mechanisms.

The most striking observation in our study was the robust upregulation of the RANTES chemokine in A172 cells. Multiple studies have shown that RANTES is upregulated following viral infection. For example, the Japanese encephalitis virus (JEV) leads to RANTES upregulation, which then induces immune cells recruitment [21]. Besides JEV, influenza virus A, Dengue-2 virus [22], respiratory syncytial virus [23], and herpes simplex virus [24] were shown to upregulate RANTES. In chronic viral infection, RANTES also plays an important role in maintaining the proper functionality of CD8+ T cells [25].

The RANTES/CCL5 chemokine is implicated in tumor progression, as well as in immune and inflammatory responses [10,26,27]. RANTES upregulation was shown to lead to cell–cell cross linking due to self-aggregation on the cell surface [10]. Identified initially as a chemoattractant protein for monocytes and T lymphocytes [28], RANTES is a ligand for CCR1 and CCR3–CCR5 receptors, with the highest affinity towards CCR5. The receptors are present on different cells, such as dendritic cells, monocytes, and mast and T cells. RANTES is produced in response to inflammatory mediators by various cells, including CD8+ T cells, fibroblasts, epithelial cells, and platelets. CCR5 receptors present on CD4+ T cells are used by human immunodeficiency virus (HIV) for entry, and RANTES serves as a protection factor against HIV entry into the host cell [29]. The RANTES gene coding sequence is localized on the 17q11.2 chromosome. This region also contains an NF1 coding sequence, the deletion of which leads to neurofibromatosis, a disease which is associated with an increased risk of developing tumors, including gliomas [30]. The duplication of the region leads to Grisart–Destree syndrome, characterized by craniofacial dysmorphism and intellectual deficits [31].

Our observations of RANTES upregulation in response to N protein in the A172 cell line point to the possible involvement of RANTES in COVID-19 disease. There are several studies suggesting a link between RANTES and SARS-CoV-2. The most interesting and relevant to our findings study showed that the N protein of SARS-CoV-2 induced RANTES in A549 cells, and it facilitated the recruitment of immune cells, such as T lymphocytes, to the site of infection, as well as promoted viral spread [32]. Besides this, Montalvo Villalba et al. demonstrated that the early stage of SARS-CoV-2 infection is characterized by an increase in interferon gamma expression and a significant decrease in TNF- β1 and RANTES expression. The suppression of RANTES during the early phase of infection may limit the migration of immune cells to the site of infection, potentially weakening the initial immune response and facilitating viral establishment [33]. There are also data showing that at later stages of the disease, RANTES levels increase by a hundredfold, especially in critically ill patients. Studies have shown that blocking CCR5 (the receptor for RANTES) with monoclonal antibodies reduces levels of inflammatory molecules such as IL-6 and interferon-related genes, leading to improved patient outcomes [34]. At the same time, in the study by Balnis et al., it was shown that a high level of RANTES in the plasma of critically ill COVID-19 patients is associated with lower mortality, which may indicate a more favorable disease outcome [35]. In line with this observation, Pérez-García F et al. demonstrated that low expression levels of the CCL5 gene and high viral load were correlated with severe cases of COVID-19 [36].

Considering all mentioned findings together with our observation, it is quite plausible to suggest A172 cells as a model system to look deeper into the potential molecular mechanisms of RANTES upregulation in response to N protein. Among several different possibilities, it is reasonable to test the involvement of interferon-regulatory factor (IRF)-3 and IRF-7, together with NF-κB transcription factor, as far as they were shown to influence RANTES expression [37]. Another intracellular pathways that should be investigated is the p38 MAPK signaling pathway, as far as Wu et al. showed that N protein induced p38 phosphorylation within A549 cells [32]. These are potential future directions that stem from our results.

Considering the chemoattractant nature of RANTES, we investigated the cell’s motility in response to N protein exposure and found that A172 cell migration is not affected by the N protein. A similar observation was reported for glial cells infected with JEV: RANTES-releasing cells did not show increased migration, while other cells exposed to glial cell supernatant demonstrated an increase in migration [21]. It would be beneficial to study whether migration is enhanced for other cells, such as monocytes, near A172 cells.

In this study, we observed that the N protein did not affect cell viability, which aligns with previous studies suggesting the antiapoptotic effect of the SARS-CoV-2 N protein [38].

As far as RANTES was upregulated in A172 cells but not in U87 cells, it is of future interest to dissect factors contributing to such cell selectivity. It is also interesting to evaluate if the N protein has any effect on RANTES in microglia and astrocytes. Lastly, it is worth investigating if the N protein of other human coronaviruses has a similar effect.

Together with RANTES, A172 cells expressed another chemokine, MCP-1, which, together with RANTES, belongs to the CC subfamily of chemokines (classification considers the position of the first two out of four conserved cysteines). Unlike RANTES, which was upregulated following N protein exposure, MCP-1 was expressed at a high level both before and after N protein stimulation. MCP-1 is a chemokine that regulates the migration of monocytes/macrophages to the site of inflammation [39].

Within this study, we also observed the upregulation of cathepsin S in A172 cells in response to 4 µg/mL of N protein. Cathepsin S is a proteolytic enzyme of lysosomal origin that is catalytically active both in acidic and neutral environments. Cathepsin S belongs to the class of cysteine proteases, and plays an important role in health and pathological conditions. Among the latter, the involvement of cathepsin S in respiratory diseases and inflammation was shown [40]. Besides this, cathepsin S also plays a role in viral entry into the host cell [41]. The spike glycoprotein of SARS-CoV-2 possesses a cleavage site that can be recognized by cathepsin S [42]. Considering the involvement of cathepsin S in respiratory diseases and in inflammation, it remains to be investigated whether the N protein of SARS-CoV-2 affects the expression of cathepsin S in lung airway epithelium, and if so, which downstream consequences are effectuated.

Whether RANTES and cathepsin S upregulation are connected to each other or not is unknown. Presently, these two observations seem to be independent findings which do require further investigation into the mechanisms involved.

In this study, we also observed the different patter of cathepsin expression in A172 and U87 cells, where unstimulated A172 cells had higher levels of expression of cathepsin V and cathepsin X compared to U87 cells, while unstimulated U87 cells demonstrated higher levels of urokinase, MMP-3, MMP-2, DPPIV, cathepsin S, and ADAMTS1 compared to A172 cells. These differences point to the fact that the cell lines are quite distinct in their physiology, despite sharing a similar origin. As far as details of such difference in protease expression in both cell lines is not thoroughly investigated, we can only hypothesize about the consequences of such differences. For example, considering involvement of urokinase in cell migration [43], we can hypothesize that a higher level of urokinase in U87 cells correlates with a higher mobility of these cells, compared to A172 cells [44]. The link between urokinase in U87 cells and the increased cell migration needs further investigation.

Multiple studies demonstrated the involvement of cathepsin L in SARS-CoV-2 pathogenesis, where cathepsin L was shown to be able to cleave viral spike glycoprotein, and thus enhanced viral entry to the host cell [45]. In our study, the level of cathepsin L in A172 and U87 cells did not change in response to the N protein.

Overall, our findings highlight the involvement of the RANTES chemokine in the cellular effect of the SARS-CoV-2 nucleocapsid protein. This finding suggests A172 cells as a model system for further investigation of the mechanisms of RANTES upregulation as well as its consequences.

## 4. Materials and Methods

### 4.1. Cell Lines and Reagents

A172 and U-87 MG cells (U87) were from ATCC (ATCC-TCP-1018 Glioma Cell Line Panel). U87 cells were cultured in EMEM media (M0894-1L, Sigma-Aldrich, St. Louis, MO, USA), A172 in DMEM media (D7777, Sigma-Aldrich) supplemented with 10% fetal bovine serum (FBS), 4 mM glutamine, 17.85 mM sodium bicarbonate (S5761, Sigma-Aldrich) and penicillin (100 units/mL)-streptomycin (100 μg/mL) (15140122, Gibco™, Grand Island, NE, USA) at 37 °C in a humidified incubator; Recombinant Severe acute respiratory syndrome coronavirus 2 Nucleoprotein (N protein) was from Cusabio (CSB-EP3325GMY).

### 4.2. Cytokine Array Assay

U87 cells were seeded in a 6-well plate at a density of 50,000 cells/cm^2^, and A172 cells were plated at 20,000 cells/cm^2^ density; after 24 h the cells, were stimulated with 1 µg/mL of recombinant N protein for 48 h. Following incubation, the supernatant was collected and applied to protein array membranes in accordance with the protocol for the Human Inflammation Antibody Array (Abcam, ab 134003, Cambridge, UK). The signal visualization was performed on the ChemiDoc™ MP Imaging System (Bio-Rad, Hercules, CA, USA). The analysis of dot intensities was performed using Image J (NIH, Bethesda, MD, USA).

### 4.3. RT-qPCR

Total RNA was isolated using the RNeasy Mini Kit from Qiagen (74104, Hilden, Germany). RNA concentration (A260 nm) and purity (A260/A280 nm) were measured using a NanoDrop 8000 Spectrophotometer (Thermo Scientific™, Waltham, MA, USA). An amount of 2 µg of total RNA was reverse-transcribed into cDNA using a Maxima First Strand cDNA Synthesis Kit (K1642, Thermo Fisher Scientific, Waltham, MA, USA). Samples of 100-fold-diluted cDNA were subjected to RT-qPCR using a reaction mixture consisting of 10 µL iTaq™ Universal SYBR^®^ Green Supermix (1725124, Bio-Rad, Hercules, CA, USA), 1 µL PCR primers (200 nM each), and Nuclease-Free Water (129115, Qiagen, Hilden, Germany). The signals were detected by the Real-Time PCR System (CFX96, Bio-Rad, Hercules, CA, USA). Thermal cycle conditions were as follows: 95 °C for 30 s, followed by 40 cycles of denaturation at 95 °C for 5 s, annealing at 54 °C (A172 cells) and 53 °C (U87 cells) for 15 s, and extension at 72 °C for 10 s, followed by the melt curve step. The expression level of each target gene was normalized to GAPD, which served as the housekeeping genes. The results were analyzed using the 2^−ΔΔCt^ method [46]. The following primers were used: RANTES/CCL5 (Regulated upon activation, normal T cell expressed and secreted), TIMP-2 (Tissue inhibitor of metalloproteinases-2), MCP-1 (Monocyte Chemoattractant Protein-1/CCL2), GAPD (Glyceraldehyde-3-phosphate dehydrogenase), IL-6 (Interleukin-6), IL-8 (Interleukin-8). The primers’ sequences are shown in Table 1.

### 4.4. Cell Viability Assay by Flow Cytometry

A172 (20,000 cells/cm^2^) and U87 (50,000 cells/cm^2^) cell lines were seeded onto a 6-well plate for 24 h. Before stimulation, the medium was replaced with a fresh one, and the cells were incubated for 48 h with the 1 µg/mL final concentration of recombinant severe acute respiratory syndrome coronavirus 2 Nucleoprotein (N). PBS served as a negative control.

Cells were washed in PBS, trypsinized, and collected into a 1 mL tube. Following this, the cells were centrifuged at 500 g for 5 min, and stained with 10 µg/mL of Propidium Iodide (PI) in the dark at 37 °C for 15 min. After incubation, cell viability assays were performed using a flow cytometer (Attune NxT Thermo Fisher, Waltham, MA, USA). Orange emission from PI was filtered through a 585/16 nm YL4 filter. The low flow rate setting (25 µL/min) was used for sample acquisition to improve the coefficient of variation. For each sample, a minimum of 50,000 events were collected within the singlet gate. All experiments were repeated at least three times. The data shown represent mean ± SD. Analyses were carried out using Microsoft Excel software.

### 4.5. Scratch Assay

A172 cells were split at 80–90% confluence, and the cells were plated in a 24-well plate at a density of 37,500 cells/cm^2^ for 12 h; the cells were then washed with PBS and incubated for 24 h with the 1 µg/mL concentrations of N protein (catalog CSB-EP3325GMY Cusabio). After 24 h, scratches were made by using 1 mL micropipette tips. Images were collected at 10 min, 12 h, 24 h, 36 h and 48 h, under the Zeiss PrimoVert Inverted Cell Culture microscope (Zeiss, Oberkochen, Germany), and the cell migration was analyzed using the Wound healing plugin for Image J software [47].

### 4.6. The Protease Profiler Array

The Proteome Profiler™ Human Protease Array Kit (Protease Profiler Array, R & D Systems, Minneapolis, MN, USA) can be used to detect up to 35 human proteases in cell lysate, in accordance with the manufacturer’s protocol. Cells were treated with 1 µg/mL or 4 µg/mL of N protein for 24 h; cells were lysed by using Lysis Buffer 17, and the protein concentration was quantified by a Bradford assay. Initially, the Protease Profiler Array membrane was equilibrated and blocked with 2 mL of Array Buffer 6 in a 4-well multi-dish for 1 h at room temperature (RT). While the blocking on the shaker was in progress, the cell lysate from A172 (1 mg) or U87 (0.5 mg) was mixed with Array Buffer 6 to a final volume of 1.5 mL, including protease inhibitors leupeptin, aprotinin, and pepstatin A (Tocris, Abingdon, UK) at a final concentration of 10 µM. Then, 15 µL of the Protease Detection Antibody Cocktail was added to the sample, mixed, and incubated for an additional hour at RT. Array Buffer 6 was aspirated from the 4-well multi-dish, and the prepared sample/antibody mixture was added to the membrane (overnight at 4 °C on a shaker). The following day, the membrane was washed three times for 10 min each time, 2 mL of Streptavidin-HRP dilution (1:2000) was added to the membrane and incubated for 30 min at RT on a shaker. After three additional washing steps, the membrane was developed with 1 mL of the Chemi Reagent Mix. Finally, the chemiluminescence was visualized using the chemiluminescence detector ChemiDoc MP (BioRad, Hercules, CA, USA), analyzed using Image Lab Touch Software 2.4.0.03 (BioRad, Hercules, CA, USA), and quantified with QuickSpots software 25.6.0.3 (Ideal Eyes Systems, Bountiful, UT, USA).

## 5. Conclusions

Within current study we tested the effect of N protein on glioblastoma A172 and U87 cell lines.

We noticed that the A172 and U87 cells show distinct profile of cytokine and protease expression. A172 cells were characterized by high levels of MCP-1 and TIMP-2 in an unstimulated state. U87 cells showed high levels of expression of IL-6, IL-8, and TIMP-2 cytokines in stimulated as well as unstimulated with N protein states. The levels of these cytokines did not change following incubation with N protein. Regarding protease level, it was noticed that uPA, MMP-3, MMP-2, ADAMTS1, and DPPIV were expressed by U87 cells, and not by A172 cells. It was also noticed that the N protein upregulated the level of cathepsin S specifically in A172 cells, but not in U87 cells. These differences in the expression profile of two cell lines point to the significant differences in the intracellular mechanisms, which require further thorough investigation. These differences could be used as markers or “fingerprints” to distinguish both cell lines. The relevance of these differences to the glioblastoma disease remains unknown at present and requires future studies.

Following the incubation with N protein, A172 demonstrated the robust upregulation of RANTES; this effect was cell-type-specific, and was not observed in U87 cells. The upregulation was detected at the protein level and at the mRNA level. At the same time, the N protein did not influence IL-6 expression in both cell lines tested. It also had no effect on A172 cell migration, and cell viability of both cell lines.

The mechanisms leading to RANTES upregulation in response to N protein in A172 cells remain to be investigated, together with cellular consequences of such upregulation.

## Figures and Tables

**Table 1:** Primer sequences.

Primers	Forward	Reverse
RANTES	CGAAAGAACCGCCAAGTGTG	GCCTCCCAAGCTAGGACAAG
TIMP-2	ACCCTCTGTGACTTCATCGTGC	GGAGATGTAGCACGGGATCATG
MCP-1	CAGCAGGTGTCCCAAAGAAGCTGT	CCATTCCTTATTGGGGTCAGCACAGA
IL-6	AGCAAAGAGGCACTGGCAGAAAACA	AGAAGAAGGAATGCCCATTAACAAC
IL-8	GAGAGTGATTGAGAGTGGACCAC	CACAACCCTCTGCACCCAGTTT
GAPD	TGCACCACCAACTGCTTAGC	GGCATGGACTGTGGTCATGAG

## Data Availability

The original contributions presented in this study are included in the article. Further inquiries can be directed to the corresponding author.

## References

[1] Naqvi A.A.T., Fatima K., Mohammad T., Fatima U., Singh I.K., Singh A., Atif S.M., Hariprasad G., Hasan G.M., Hassan M.I. (2020). Insights into SARS-CoV-2 Genome, Structure, Evolution, Pathogenesis and Therapies: Structural Genomics Approach. Biochim. Biophys. Acta Mol. Basis Dis..

[2] Wang X., Tang G., Liu Y., Zhang L., Chen B., Han Y., Fu Z., Wang L., Hu G., Ma Q. (2022). The Role of IL-6 in Coronavirus, Especially in COVID-19. Front. Pharmacol..

[3] Zhang X., Wu K., Wang D., Yue X., Song D., Zhu Y., Wu J. (2007). Nucleocapsid Protein of SARS-CoV Activates Interleukin-6 Expression through Cellular Transcription Factor NF-κB. Virology.

[4] Karwaciak I., Sałkowska A., Karaś K., Dastych J., Ratajewski M. (2021). Nucleocapsid and Spike Proteins of the Coronavirus SARS-CoV-2 Induce IL6 in Monocytes and Macrophages-Potential Implications for Cytokine Storm Syndrome. Vaccines.

[5] Mao L., Jin H., Wang M., Hu Y., Chen S., He Q., Chang J., Hong C., Zhou Y., Wang D. (2020). Neurologic Manifestations of Hospitalized Patients With Coronavirus Disease 2019 in Wuhan, China. JAMA Neurol..

[6] Helms J., Kremer S., Merdji H., Clere-Jehl R., Schenck M., Kummerlen C., Collange O., Boulay C., Fafi-Kremer S., Ohana M. (2020). Neurologic Features in Severe SARS-CoV-2 Infection. N. Engl. J. Med..

[7] Brola W., Wilski M. (2022). Neurological Consequences of COVID-19. Pharmacol. Rep. PR.

[8] Tsukahara T., Brann D.H., Datta S.R. (2023). Mechanisms of SARS-CoV-2-Associated Anosmia. Physiol. Rev..

[9] Meinhardt J., Radke J., Dittmayer C., Franz J., Thomas C., Mothes R., Laue M., Schneider J., Brünink S., Greuel S. (2021). Olfactory Transmucosal SARS-CoV-2 Invasion as a Port of Central Nervous System Entry in Individuals with COVID-19. Nat. Neurosci..

[10] Appay V., Rowland-Jones S.L. (2001). RANTES: A Versatile and Controversial Chemokine. Trends Immunol..

[11] Palesch D., Wagner J., Meid A., Molenda N., Sienczyk M., Burkhardt J., Münch J., Prokop L., Stevanovic S., Westhoff M.-A. (2016). Cathepsin G-Mediated Proteolytic Degradation of MHC Class I Molecules to Facilitate Immune Detection of Human Glioblastoma Cells. Cancer Immunol. Immunother. CII.

[12] Fajgenbaum D.C., June C.H. (2020). Cytokine Storm. N. Engl. J. Med..

[13] Montazersaheb S., Hosseiniyan Khatibi S.M., Hejazi M.S., Tarhriz V., Farjami A., Ghasemian Sorbeni F., Farahzadi R., Ghasemnejad T. (2022). COVID-19 Infection: An Overview on Cytokine Storm and Related Interventions. Virol. J..

[14] Karki R., Sharma B.R., Tuladhar S., Williams E.P., Zalduondo L., Samir P., Zheng M., Sundaram B., Banoth B., Malireddi R.K.S. (2021). Synergism of TNF-α and IFN-γ Triggers Inflammatory Cell Death, Tissue Damage, and Mortality in SARS-CoV-2 Infection and Cytokine Shock Syndromes. Cell.

[15] Elbadawy H.M., Khattab A., El-Agamy D.S., Eltahir H.M., Alhaddad A., Aljohani F.D., Almuzaini T.M., Abouzied M.M., Aldhafiri A. (2023). IL-6 at the Center of Cytokine Storm: Circulating Inflammation Mediators as Biomarkers in Hospitalized COVID-19 Patients. J. Clin. Lab. Anal..

[16] Huang C., Wang Y., Li X., Ren L., Zhao J., Hu Y., Zhang L., Fan G., Xu J., Gu X. (2020). Clinical Features of Patients Infected with 2019 Novel Coronavirus in Wuhan, China. Lancet.

[17] Hojyo S., Uchida M., Tanaka K., Hasebe R., Tanaka Y., Murakami M., Hirano T. (2020). How COVID-19 Induces Cytokine Storm with High Mortality. Inflamm. Regen..

[18] Coomes E.A., Haghbayan H. (2020). Interleukin-6 in Covid-19: A Systematic Review and Meta-Analysis. Rev. Med. Virol..

[19] Chen X., Zhao B., Qu Y., Chen Y., Xiong J., Feng Y., Men D., Huang Q., Liu Y., Yang B. (2020). Detectable Serum Severe Acute Respiratory Syndrome Coronavirus 2 Viral Load (RNAemia) Is Closely Correlated with Drastically Elevated Interleukin 6 Level in Critically Ill Patients With Coronavirus Disease 2019. Clin. Infect. Dis. Off. Publ. Infect. Dis. Soc. Am..

[20] Zhang B., Tian J., Zhang Q., Xie Y., Wang K., Qiu S., Lu K., Liu Y. (2022). Comparing the Nucleocapsid Proteins of Human Coronaviruses: Structure, Immunoregulation, Vaccine, and Targeted Drug. Front. Mol. Biosci..

[21] Chen C.J., Liao S.L., Kuo M.D., Wang Y.M. (2000). Astrocytic Alteration Induced by Japanese Encephalitis Virus Infection. Neuroreport.

[22] Lin Y.L., Liu C.C., Chuang J.I., Lei H.Y., Yeh T.M., Lin Y.S., Huang Y.H., Liu H.S. (2000). Involvement of Oxidative Stress, NF-IL-6, and RANTES Expression in Dengue-2-Virus-Infected Human Liver Cells. Virology.

[23] Casola A., Burger N., Liu T., Jamaluddin M., Brasier A.R., Garofalo R.P. (2001). Oxidant Tone Regulates RANTES Gene Expression in Airway Epithelial Cells Infected with Respiratory Syncytial Virus. Role in Viral-Induced Interferon Regulatory Factor Activation. J. Biol. Chem..

[24] Melchjorsen J., Paludan S.R. (2003). Induction of RANTES/CCL5 by Herpes Simplex Virus Is Regulated by Nuclear Factor Kappa B and Interferon Regulatory Factor 3. J. Gen. Virol..

[25] Crawford A., Angelosanto J.M., Nadwodny K.L., Blackburn S.D., Wherry E.J. (2011). A Role for the Chemokine RANTES in Regulating CD8 T Cell Responses during Chronic Viral Infection. PLoS Pathog..

[26] Zeng Z., Lan T., Wei Y., Wei X. (2022). CCL5/CCR5 Axis in Human Diseases and Related Treatments. Genes Dis..

[27] Mrowietz U., Schwenk U., Maune S., Bartels J., Küpper M., Fichtner I., Schröder J.-M., Schadendorf D. (1999). The Chemokine RANTES Is Secreted by Human Melanoma Cells and Is Associated with Enhanced Tumour Formation in Nude Mice. Br. J. Cancer.

[28] Schall T.J., Bacon K., Toy K.J., Goeddel D.V. (1990). Selective Attraction of Monocytes and T Lymphocytes of the Memory Phenotype by Cytokine RANTES. Nature.

[29] Gross E., Amella C.A., Pompucci L., Franchin G., Sherry B., Schmidtmayerova H. (2003). Macrophages and Lymphocytes Differentially Modulate the Ability of RANTES to Inhibit HIV-1 Infection. J. Leukoc. Biol..

[30] Carrion A.W., Shah A.C., Kotch C. (2023). Neurofibromatosis Type 1-Associated Gliomas and Other Tumors: A New Pathway Forward?. Pediatr. Hematol. Oncol. J..

[31] Tassano E., Giacomini T., Severino M., Gamucci A., Fiorio P., Gimelli G., Ronchetto P. (2017). Characterization of the Phenotype Associated with Microduplication Reciprocal to NF1 Microdeletion Syndrome. Cytogenet. Genome Res..

[32] Wu J.-L., Kuan I.-I., Guo J.-Y., Hsu W.-C., Tang W.-C., Chan H.-J., Chen Y.-J., Chen B.-C., Wu H.-C., Liao J.C. (2023). SARS-CoV-2 N Protein Mediates Intercellular Nucleic Acid Dispersion, a Feature Reduced in Omicron. iScience.

[33] Montalvo Villalba M.C., Valdés Ramírez O., Muné Jiménez M., Arencibia Garcia A., Martinez Alfonso J., González Baéz G., Roque Arrieta R., Rosell Simón D., Alvárez Gainza D., Sierra Vázquez B. (2020). Interferon Gamma, TGF-Β1 and RANTES Expression in Upper Airway Samples from SARS-CoV-2 Infected Patients. Clin. Immunol..

[34] Patterson B.K., Seethamraju H., Dhody K., Corley M.J., Kazempour K., Lalezari J., Pang A.P.S., Sugai C., Mahyari E., Francisco E.B. (2021). CCR5 Inhibition in Critical COVID-19 Patients Decreases Inflammatory Cytokines, Increases CD8 T-Cells, and Decreases SARS-CoV2 RNA in Plasma by Day 14. Int. J. Infect. Dis. IJID Off. Publ. Int. Soc. Infect. Dis..

[35] Balnis J., Adam A.P., Chopra A., Chieng H.C., Drake L.A., Martino N., Bossardi Ramos R., Feustel P.J., Overmyer K.A., Shishkova E. (2021). Unique Inflammatory Profile Is Associated with Higher SARS-CoV-2 Acute Respiratory Distress Syndrome (ARDS) Mortality. Am. J. Physiol.-Regul. Integr. Comp. Physiol..

[36] Pérez-García F., Martin-Vicente M., Rojas-García R.L., Castilla-García L., Muñoz-Gomez M.J., Hervás Fernández I., González Ventosa V., Vidal-Alcántara E.J., Cuadros-González J., Bermejo-Martin J.F. (2022). High SARS-CoV-2 Viral Load and Low CCL5 Expression Levels in the Upper Respiratory Tract Are Associated with COVID-19 Severity. J. Infect. Dis..

[37] Génin P., Algarté M., Roof P., Lin R., Hiscott J. (2000). Regulation of RANTES Chemokine Gene Expression Requires Cooperativity Between NF-κB and IFN-Regulatory Factor Transcription Factors. J. Immunol..

[38] Pan P., Ge W., Lei Z., Luo W., Liu Y., Guan Z., Chen L., Yu Z., Shen M., Hu D. (2023). SARS-CoV-2 N Protein Enhances the Anti-Apoptotic Activity of MCL-1 to Promote Viral Replication. Signal Transduct. Target. Ther..

[39] Deshmane S.L., Kremlev S., Amini S., Sawaya B.E. (2009). Monocyte Chemoattractant Protein-1 (MCP-1): An Overview. J. Interferon Cytokine Res. Off. J. Int. Soc. Interferon Cytokine Res..

[40] Smyth P., Sasiwachirangkul J., Williams R., Scott C.J. (2022). Cathepsin S (CTSS) Activity in Health and Disease—A Treasure Trove of Untapped Clinical Potential. Mol. Aspects Med..

[41] Scarcella M., d’Angelo D., Ciampa M., Tafuri S., Avallone L., Pavone L.M., De Pasquale V. (2022). The Key Role of Lysosomal Protease Cathepsins in Viral Infections. Int. J. Mol. Sci..

[42] Bollavaram K., Leeman T.H., Lee M.W., Kulkarni A., Upshaw S.G., Yang J., Song H., Platt M.O. (2021). Multiple Sites on SARS-CoV-2 Spike Protein Are Susceptible to Proteolysis by Cathepsins B, K, L, S, and V. Protein Sci..

[43] Carlin S.M., Resink T.J., Tamm M., Roth M. (2005). Urokinase Signal Transduction and Its Role in Cell Migration. FASEB J. Off. Publ. Fed. Am. Soc. Exp. Biol..

[44] Kaphle P., Li Y., Yao L. (2019). The Mechanical and Pharmacological Regulation of Glioblastoma Cell Migration in 3D Matrices. J. Cell. Physiol..

[45] Zhao M.-M., Yang W.-L., Yang F.-Y., Zhang L., Huang W.-J., Hou W., Fan C.-F., Jin R.-H., Feng Y.-M., Wang Y.-C. (2021). Cathepsin L Plays a Key Role in SARS-CoV-2 Infection in Humans and Humanized Mice and Is a Promising Target for New Drug Development. Signal Transduct. Target. Ther..

[46] Livak K.J., Schmittgen T.D. (2001). Analysis of Relative Gene Expression Data Using Real-Time Quantitative PCR and the 2^−ΔΔCT^ Method. Methods.

[47] Suarez-Arnedo A., Torres Figueroa F., Clavijo C., Arbeláez P., Cruz J.C., Muñoz-Camargo C. (2020). An Image J Plugin for the High Throughput Image Analysis of in Vitro Scratch Wound Healing Assays. PLoS ONE.

